# A preliminary study on the possibility of fermented pineapple peel residue partially replacing whole corn silage in feeding Chuanzhong black goats

**DOI:** 10.3389/fmicb.2022.959857

**Published:** 2022-11-11

**Authors:** Chuang Yang, Weiran Zhao, Hanchen Tian, Mingyue Wang, Chongya Gao, Yongqing Guo, Baoli Sun

**Affiliations:** College of Animal Science, South China Agricultural University, Guangzhou, China

**Keywords:** agricultural by-products, fermented feeds, Chuanzhong black goats, pineapple peel, rumen microfora, fecal microfora, meat quality

## Abstract

This study aims to assess the effects of the partial replacement of whole corn silage (WCS) with fermented pineapple peel residue (FPPR) on growth, serological parameters, muscle quality, rumen microorganisms, and fecal microorganisms. A total of 24 Chuanzhong black goats weighing 10.23 ± 1.42 kg were evaluated in a randomized complete trial design in accordance with the following treatments: (1) 0% FPPR in the diet, (2) 25% FPPR in the diet, and (3) 50% FPPR in the diet. In goats, the partial substitution of FPPR for WCS increased the abundance of probiotics, such as *Blautia, Butyrivibrio fibrisolvens*, and *Ruminococcus albus*, and did not exert significant effects on overall serological parameters and muscle quality. In conclusion, the partial substitution of FPPR for WCS in the diet did not impair or affect the productive performance of goats.

## Introduction

Roughages are essential for ruminant production because they stimulate the chewing response and enhance saliva output to stabilize the rumen microbial environment (Tafaj et al., 2006). In Chinese rangelands, corn silage is the most common source of roughage for ruminants; however, continuous maize monoculture can negatively affect nutrient extraction from the soil and increase the chance of erosion (Silva et al., 2021). In addition, there is an urgent need to develop new sources of roughage for the progression of ruminant farming in many areas where maize cultivation is unsuitable due to climatic, geographical, or economic conditions.

Pineapple, which is widely distributed in the tropics, is well received by consumers for its unique fragrance and rich nutrition. Moreover, it promotes the body's health (Brito et al., 2020; Padrón-Mederos et al., 2020). In addition to being consumed fresh, pineapple is often used to produce fruit juices, jams, dried fruits, and syrups (Sanya et al., 2020). However, the edible part of pineapple only accounts for 30–45% of its total weight, and the rest parts are often discarded or buried in landfills, causing great environmental pollution and resource waste (Gasmi Benahmed et al., 2021). Pineapple peel residue (PPR) is a by-product of pineapple processing that is rich in nutrients and bioactive substances (Roda and Lambri, 2019). This waste is mainly composed of dietary fibers, flavonoids, minerals, sugars, polyphenols, and vitamins and plays an important role in antibiosis, antioxidation, and gastrointestinal protection (Hu et al., 2019). Therefore, it can be developed as a feed material. Studies on the use of pineapple residue as feed have been reported. These works demonstrated that in animals, the favorable palatability of pineapple residue can improve feed intake (Shivakumar Gowda et al., 2015; Kiggundu and Kabi, 2019; Liu et al., 2021) and that PPR has no adverse effects on health and metabolism (Shivakumar Gowda et al., 2015; Kiggundu and Kabi, 2019).

Rumen microorganisms can transform fibrous materials into energy needed for daily activities of ruminants and execute important roles in regulating host physiological functions, metabolic reactions, and resistance to pathogens (Jami et al., 2013; Wang et al., 2013). Bacteria dominate rumen microorganisms and play a crucial role in the digestion and conversion of feed into short-chain fatty acids and mycoproteins (Brulc et al., 2009). Diet composition is an important factor affecting rumen microorganisms. A previous study found that in lambs, substituting a certain proportion of buckwheat straw for corn straw in the feed reduced the diversity of rumen microflora. Another study (Wang et al., 2021) reported that grapeseed decreased *Firmicutes* and increased *Bacteroidetes* and *Prevotella_1* in the rumen microflora of lambs. Nevertheless, studies reporting the effects of PPR on the rumen microflora of ruminants are limited.

Intestinal microorganisms also perform an important role in host health, and the disorder of microbial composition may lead to inflammation and diseases (Lee et al., 2020). Intestinal flora is affected by numerous factors, such as physiological status, nutrient composition, environmental changes, drug treatments, pathogens, and stress (Kunz et al., 2019). In animal husbandry, numerous works have demonstrated that different feed ingredients cause changes in the composition of fecal microbiota (Sun et al., 2017; Xie et al., 2020). In our previous study, we found that replacing whole corn silage (WCS) with a certain proportion of *Broussonetia papyrifera* silage could affect the composition and function of fecal microbiota in Holstein heifers (Tian et al., 2020). However, related studies on the effects of fermented PPR (FPPR) as a new feed material on the intestinal microflora in ruminants are still limited.

Although FPPR has the potential to replace corn silage as a new source of roughage due to its nutritional richness, there is a lack of the literature assessing its feasibility in ruminant feeding. This study aims to use FPPR as a partial replacement for maize silage as a source of roughage for Chuanzhong black goats and to evaluate growth performance, serological parameters, muscle quality, rumen microbiology, and fecal microbiology for complementing the theoretical basis to determine the feasibility of FPPR as a source of roughage for ruminants.

## Materials and methods

All experimental procedures used in this study were approved by the Committee of Animal Experiments of South China Agricultural University (No. 2021G018).

### Experimental materials

Pineapple peel residue was purchased from a technology company in Leizhou city. After being crushed and pressed, the PPR measurement was approximately 2–3 cm. Then, it was evenly sprayed with a commercial starter (moisture ≤ 12%; *Bacillus subtilis* ≥ 2 × 10^9^ cfu/g; *Peeltococcus acitilactict* ≥ 2 × 10^9^ cfu/g; and *Candida utilis* ≥ 4 × 10^9^ cfu/g) solution. Approximately 40 kg of PPR was sealed into a polyethylene fermentation bag, and after 20 days of fermentation, FPPR was obtained.

### Determination of nutrients in diets

The nutrient composition of the feed used in the experiment was determined as follows: dry matter, crude protein, ether extract, and ash were measured based on the method described by the Association of Official Analytical Chemists (2002). Neutral detergent fiber and acid detergent fiber were determined in accordance with a previous study (Van Soest et al., 1991). Calcium and phosphorus were tested *via* the ethylenediaminetetraacetic acid (EDTA) complexometric titration method and vanadium molybdate yellow colorimetric method, respectively.

### Experimental animals

The trial was conducted at a commercial farm in Zhaoqing (Guangdong province, China). It included a preliminary feeding period of 7 days and a formal experimental period of 35 days. A total of 24 healthy 4-month-old female Chuanzhong black goats (10.23 ± 1.42 kg) with similar genetic backgrounds were evenly and randomly assigned into three groups. In all three groups, the substitution amounts of FPPR for WCS in the diet were 0% (CON), 25% (B25), and 50% (B50). The diets were mixed in accordance with the National Research Council (2016), and their nutrient compositions were analyzed according to Xie et al. (2020). The ingredients and nutrient composition of these diets are shown in Table 1. Feeding was performed at 09:00 and 17:00 every day, and the goats were allowed to eat and drink freely during the experiment. During the experimental period, feed intake and average daily gain (ADG) were measured.

**Table 1:** The ingredients and chemical composition of the diets.

**Item^a^**	**Diets treatments** ^**b**^		
	**CON**	**B25**	**B50**
Ingredient (as fed-basis %)			
WCS	76.20	57.20	38.10
FPPR	0.00	19.00	38.10
Peanut seedling hay	19.00	19.00	19.00
Indian meal	2.93	2.93	2.93
Soybean meal	1.20	1.20	1.20
Alfalfa hay	0.47	0.47	0.47
Calcium hydrophosphate	0.05	0.05	0.05
Mountain flour	0.10	0.10	0.10
Salt	0.05	0.05	0.05
Nutrient content			
DM (%)	30.84	30.39	30.34
CP (%/DM)	11.68	11.99	11.45
EE (%/DM)	0.01	0.02	0.02
Ash (%/DM)	0.13	0.12	0.12
NDF (%/DM)	58.11	52.98	54.29
ADF (%/DM)	36.20	33.35	33.12
Ca (%/DM)	0.90	0.97	0.97
P (%/DM)	0.44	0.42	0.42
NEm (mcal/kg)	1.18	1.25	1.29
NEg (macl/kg)	0.62	0.69	0.72

a WCS, whole corn silage; FPPR, fermented pineapple peel residue; DM, dry matter; CP, crude protein; EE, ether extract; NDF, neutral detergent fiber; ADF, acid detergent fiber; NEm, net energy for maintenance; NEg, net energy for gain.

b CON, FPPR replaces 0% of WCS; B25, FPPR replaces 25% of WCS; B50, FPPR replaces 50% of WCS.

### Collection of samples

Serum sample collection was carried out on days 17 and 35 of the formal experimental period. Goats were fasted for 12 h before blood sampling, and blood was collected from the jugular vein. After centrifugation at 4,000 r/min for 15 min, the upper serum was removed and stored at −20°C.

Fecal samples were collected from the rectum on the last day of the experimental period. Approximately 2 g of the sample was stored in a 2-ml frozen pipe at −80°C for the determination of bacterial communities.

A total of 16 goats from CON and B50, which showed a strong effect of FPPR on ADG and feed intake, were selected and mercy killed on the last day of the experimental period. The rumen fluid from each goat was filtered through the four layers of sterile gauze, and 150 ml was collected. Filtered rumen fluid was poured into a 2-ml frozen pipe and 50-ml centrifuge tube. The samples in the frozen pipes were stored at −80°C for the analysis of bacterial community. The pH value of the rumen fluid in the centrifuge tube was determined using a pH meter (FE28-Standard, METTLER-TOLEDO). Then, the rumen fluid was centrifuged at 5,000 r/min for 15 min. The supernatant obtained through centrifugation was removed and stored at −20°C for the determination of fermentation parameters.

The longissimus dorsi muscles of the killed goats were removed and divided into two parts along their sections. One was used for the determination of pH, meat color (brightness, L^*^; redness, a^*^; and yellowness, b^*^), drip loss, shear force, and water-holding capacity (WHC) through the determination methods reported by Abdel-Wareth et al. (2019) and Wassie et al. (2020). Another part was used for the determination of amino acid content in muscles with an L-8900 amino acid analyzer (Hitachi, Japan) in accordance with the method of Fu et al. (2016).

### Determination of serum indicators

Serum biochemical indicators, including albumin, total protein, globulin, uric acid, blood urea (UREA), nonesterified fatty acids (NEFA), creatine kinase, and blood glucose, were determined using a HITACHI Automatic Analyzer 7600 (Hitachi, Ltd., Tokyo, Japan). Interferon-γ, C-reactive protein, β-hydroxybutyric acid, heat shock protein-70, triiodothyronine, and immunoglobulin family were measured *via* the ELISA method. Catalase, glutathione peroxidase, and total antioxidant capacity were measured using commercial colorimetric ABTS kits (Nanjing Jiancheng Institute of Bioengineering, Nanjing).

### Analysis of rumen and fecal bacterial communities

The cetyltrimethyl ammonium bromide method was adopted to extract genomic deoxyribonucleic acid (DNA) from the samples. The genomic DNA sample were tested for purity, concentration, and integrity. The 16S rDNA V1–V9 regions were amplified with a tansStart^®^ FastPfu DNA polymerase kit. The forward and reverse primers were designed as 27F (GAGAGTTTGATCCTGGCTCAG) and 1541R (AAGGAGGTGATCCAGCCGCA), respectively (Jin et al., 2018). Reaction systems were established as follows: Trans Fastpfu, 1 μl; 5× buffer, 10 μl; 5× Stimulate, 5 μl; dNTP, 5 μl; forward primer, 1 μl; reverse primer, 1 μl; gDNA 5 μl; and nuclease-free water, 23 μl. The reaction conditions were set as follows: initial denaturation, 95°C for 2 min; denaturation (95°C, 30 s), annealing (60°C, 45 s), and extension (72°C, 90 s) for 35 cycles; and final extension, 72°C for 10 min. After amplification, the quality and integrity of the PCR products were determined by electrophoresis with 2% agarose gel. Then, the products were purified using a QIAquick Gel Extraction kit (QIAGEN, Inc., Valencia, CA, USA). The sequencing library was generated with a SMRTbellTM template preparation kit (Pacific Biosciences, Inc., Menlo Park, CA, USA) (Song et al., 2019). Library quality was evaluated by using a Qubit^@^ 2.0 fluorimeter and FEMTO Pulse system. Finally, the PacBio Sequel platform (Pacific Biosciences, Inc., Menlo Park, CA, USA) was selected for library sequencing, and the data were subjected to bioinformatics analyses based on the methods described by Yang et al. (2020). α-diversity, including Ace, Chao1, Shannon, and Simpson indexes, was calculated using Qiime software, and β-diversity was analyzed with the “vegan” package in R software (De Filippis et al., 2018). Functional prediction of the microflora was performed using Tax4Fun (Sharma et al., 2020). Linear discriminant analysis (LDA) effect size (LEfSe) and functional prediction of the microflora were carried out using an online tool (http://huttenhower.sph.harvard.edu/galaxy/), and *p* < 0.05 and LDA score >2 were selected as cutoffs (Cadena et al., 2019).

## Statistical analysis

Test data were preliminarily sorted and analyzed using Excel software (Microsoft, Redmond, WA, USA) and then analyzed with SAS 9.4 software (SAS Institute, Inc., Cary, NC, USA). The normality of the data was tested using the UNIVARIATE procedure. Outliers were processed based on the Studentized absolute residual values > 2.5.

The MIXED procedure model used for the data from meat quality processing is *Y*_*ij*_ = μ + *B*_*i*_ + *T*_*j*_ + ε_*i*_, where *Y*_*ij*_ is the value of the dependent variable of the test goats under different treatments, μ is the overall mean, *B*_*i*_ are the dietary treatment effects, *T*_*j*_ are the time treatment effects, and ε_*ij*_ is the random error.

The generalized linear model (GLM) procedure model used for data other than meat quality processing data is *Y*_*i*_ = μ + *B*_*i*_ + ε_*i*_, where *Y*_*i*_ is the value of the dependent variable of the test goats under different treatments, μ is the overall mean, *B*_*i*_ are the dietary treatment effects, and ε_*i*_ is the random error.

Unless otherwise noted, test data are shown in figures and tables as mean and standard deviation (SD), and *p* < 0.05 indicates a significant difference.

## Results

### Performance and serum indicators

**Figure 1:**
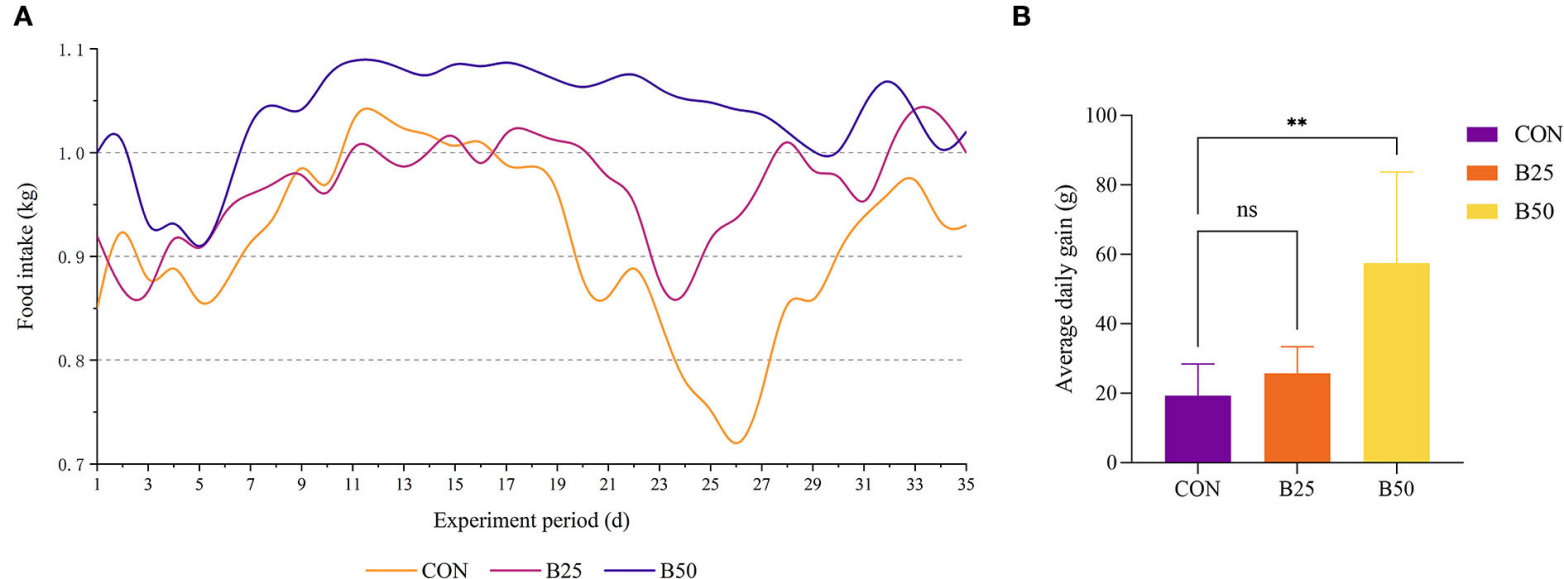
Effect of fermented pineapple peel residue (FPPR) on the growth performance of black goats. **(A)** Feed intake and **(B)** average daily gain (ADG). **p < 0.01; ns, No Significant.

**Figure 2:**
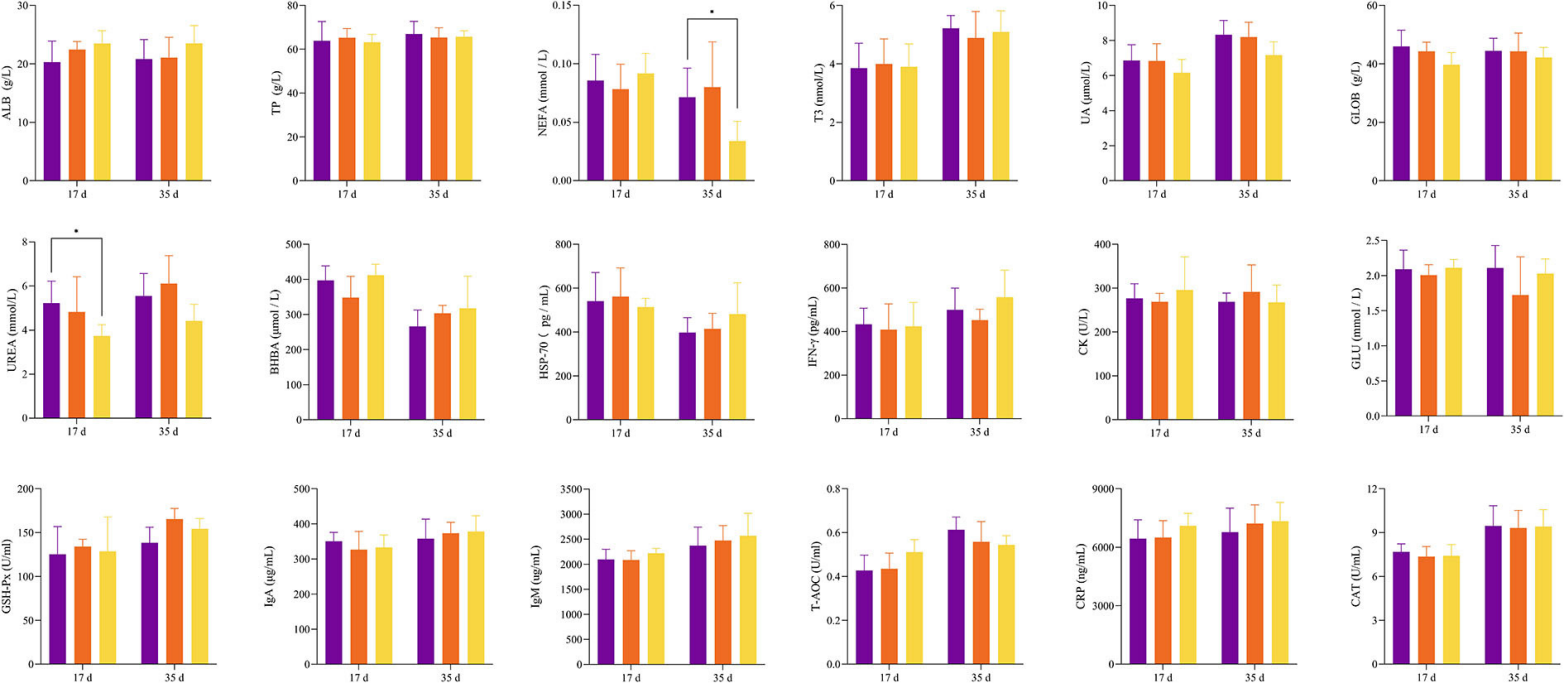
Effect of FPPR on the serum indicators of black goats.

Throughout the experiment, the feed intake of the CON group was always lower than that of the B50 group (Figure 1A) and the ADG of B50 was significantly higher than that of CON (*p* < 0.05). However, the B25 group did not significantly differ from the CON group (*p* > 0.05) (Figure 1B). Figure 2 shows that on day 17, the UREA in B50 was significantly lower than that in CON (*p* < 0.05). On day 35, NEFA in B50 was significantly lower than that in CON (*p* < 0.05). The contents of other serum indicators did not differ significantly among all groups.

### Meat quality

The results of meat quality and amino acid composition of longissimus dorsi in goats are shown in Tables 2, 3. The physical properties presented in Table 2 indicated that FPPR decreased the pH value of longissimus dorsi at 45 min and 48 h (*p* < 0.05). The results in Table 3 demonstrated that FPPR reduced the content of phenylalanine (*p* < 0.05), whereas the contents of total amino acids (TAA), essential amino acids (EAA), and delicious amino acids (DAA) did not change significantly (*p* > 0.05).

**Table 2:** Effect of the partial substitution of fermented pineapple peel residue (FPPR) for WSC on the meat quality of longissimus dorsi in Chuangzhong black goats.

**Items^a^**		**Treatments**		***P*-value**
		**CON**	**FPPR**	
	45 min	6.05 ± 0.10	5.62 ± 0.12	0.002
pH	24 h	5.80 ± 0.09	5.68 ± 0.07	0.096
	48 h	6.03 ± 0.05	5.81 ± 0.06	0.002
	45 min	55.14 ± 2.82	54.63 ± 2.66	0.796
L*	24 h	53.60 ± 3.78	52.00 ± 2.18	0.483
	48 h	54.25 ± 1.81	54.40 ± 1.24	0.889
	45 min	15.45 ± 1.17	14.22 ± 0.81	0.123
a*	24 h	15.99 ± 2.00	14.18 ± 0.99	0.143
	48 h	16.15 ± 1.14	15.01 ± 1.86	0.325
	45 min	8.56 ± 0.85	8.10 ± 0.77	0.438
b*	24 h	10.05 ± 0.89	9.02 ± 0.59	0.145
	48 h	9.97 ± 0.89	9.15 ± 1.70	0.858
DL (%)		21.12 ± 5.23	17.48 ± 4.55	0.365
SF (N)		51.36 ± 4.51	50.02 ± 1.19	0.581
WHC (%)		94.26 ± 1.42	92.63 ± 1.71	0.182

a DL, drip loss; SF, shear force; WHC, water-holding capacity.

**Table 3:** Effect of the partial substitution of FPPR for WSC on the amino acid composition of longissimus dorsi in Chuangzhong black goats (g/100 g protein).

**Items^a^**	**Treatments**		***P*-value**
	**CON**	**FPPR**	
Alanine^$^	4.35 ± 0.12	4.37 ± 0.19	0.844
Arginine^#^	5.04 ± 0.16	4.94 ± 0.17	0.468
Aspartic acid^$^	6.85 ± 0.36	6.63 ± 0.25	0.372
Cysteine	0.78 ± 0.16	0.64 ± 0.09	0.185
Glutamic acid^$^	11.65 ± 0.66	11.04 ± 0.61	0.241
Glycine^$^	3.47 ± 0.11	4.24 ± 0.83	0.149
Histidine^#^	1.92 ± 0.40	2.40 ± 0.25	0.091
Isoleucine^#^	3.54 ± 0.18	3.36 ± 0.19	0.235
Leucine^#^	6.36 ± 0.23	5.95 ± 0.25	0.061
Lysine^#^	6.95 ± 0.27	6.48 ± 0.29	0.064
Methionine^#^	1.99 ± 0.16	1.91 ± 0.10	0.465
Phenylalanine^#, $^	3.20 ± 0.18	2.95 ± 0.07	0.041
Proline	3.08 ± 0.08	3.34 ± 0.43	0.335
Serine	2.88 ± 0.12	2.79 ± 0.11	0.322
Threonine^#^	3.50 ± 0.16	3.33 ± 0.14	0.170
Tyrosine^$^	2.70 ± 0.28	2.45 ± 0.11	0.149
Valine^#^	3.78 ± 0.17	3.61 ± 0.14	0.198
EAA	36.28 ± 1.77	34.94 ± 1.38	0.297
DAA	32.23 ± 1.20	31.69 ± 0.99	0.536
TAA	72.04 ± 2.90	70.44 ± 1.90	0.408

a EAA, essential amino acids, including Arginine, Histidine, Isoleucine, Leucine, Lysine, Methionine, Phenylalanine, Threonine, and Valine, marked with “#” as a superscript in the table; DAA, delicious amino acids, including Alanine, Aspartic acid, Glutamic acid, Glycine, Phenylalanine, and Tyrosine, marked with “$” as a superscript in the table; TAA, total amino acids. Asparagine, Glutamine, and Tryptophane were undetected in this study.

### The diversity of fecal and rumen fluid microflora

**Figure 3:**
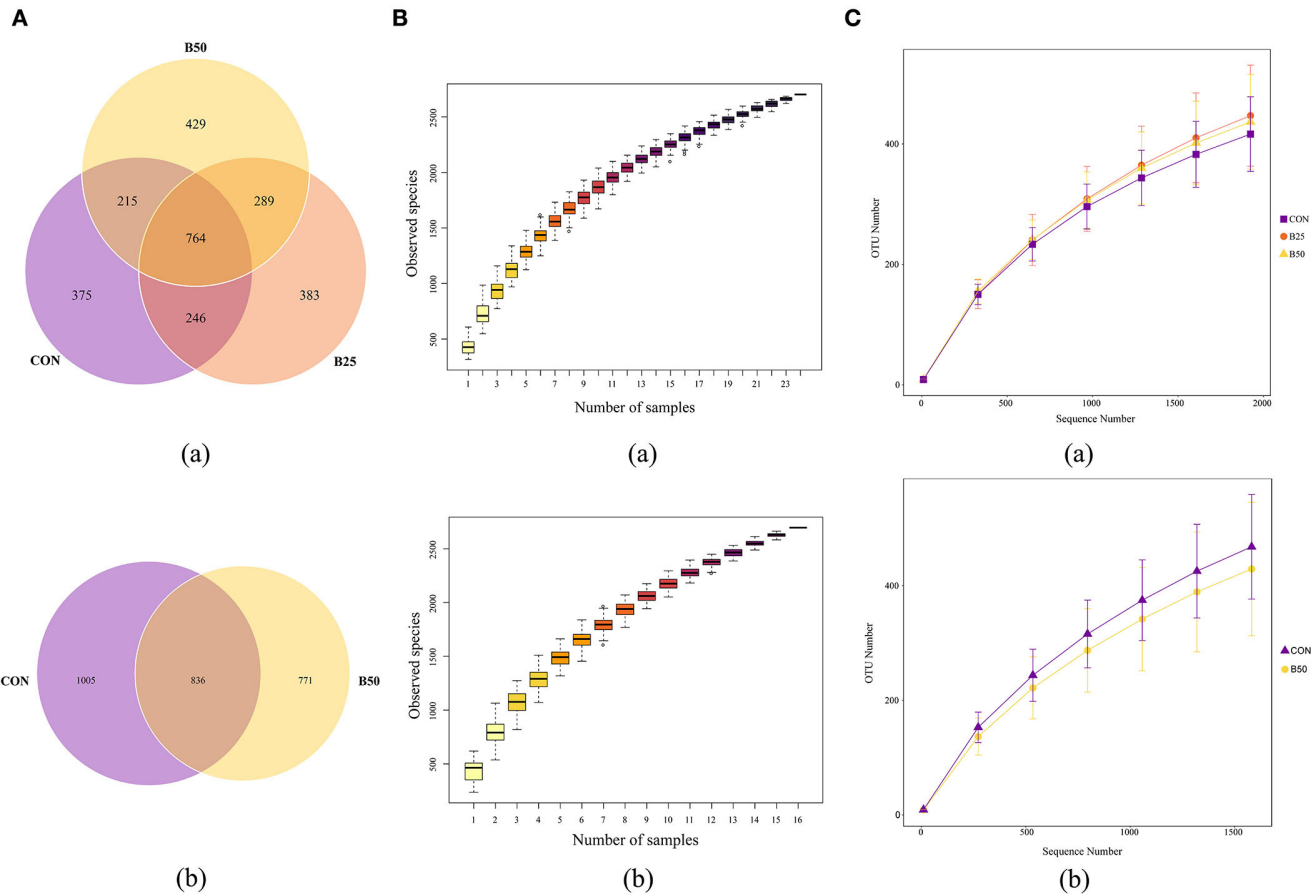
Overall microbial species composition **(A)** a Venn diagram of operational taxonomic units (OTUs); **(B)** species accumulation boxplot; and **(C)** rarefaction curve.

The 24 fecal samples were sequenced using the PacBio platform, and 278,008 raw read sequences were obtained. After removing primers and mosaics, 193,765 optimized sequences were retained. The optimized sequences were 1,432–1,441 bp in length and had an average length of 1,438 bp. The 16 samples of rumen fluid from CON and B50 were also sequenced using the PacBio platform, and 233,489 raw reads and 170,176 clean reads were obtained. The average lengths of these valid sequences were 1,429–1,477 nt. Clean reads from all samples were clustered to study the diversity of the species composition of the samples, and their sequences were grouped into operational taxonomic units (OTUs) with 97% identity. Then, species annotation was performed on the representative sequences of OTUs. A total of 2,701 and 2,612 effective OTUs were obtained from fecal and rumen fluid samples, respectively. A Venn diagram was used to illustrate the composition of unique and shared fecal and rumen fluid species. The Venn diagram of the fecal samples (Figure 3Aa) showed that there were 764 OTUs under all the three treatments; 375, 383, and 429 OTUs were exclusive to CON, B25, and B50, respectively. In the rumen fluid, 836 OTUs were present in both treatments (Figure 3Ab), and 1,005 and 771 OTUs were exclusively present in the CON and B50 treatments, respectively. The observed species curve (Figure 3B) and species accumulation boxplot (Figure 3C) show that with increasing sequence numbers, the curves tended to become level gradually, indicating that sampling was sufficient for data analysis.

**Figure 4:**
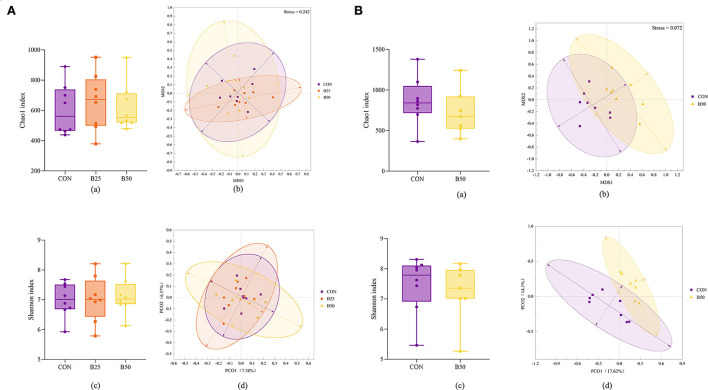
α- and β-diversity of fecal and rumen fluid microflora affected by FPPR. **(A)** α- and β-diversity for fecal microflora: (a) Chao1 index; (b) non-metric multidimensional scaling (NMDS) plot; (c) Shannon index; (d) principal co-ordinates analysis (PCoA) plot; **(B)** α- and β-diversity for rumen fluid microflora: (a) Chao1 index; (b) NMDS plot; (c) Shannon index; (d) PCoA plot.

The results of the α-diversity of fecal and rumen fluid microflora (Figures 4A,B, a,c) were obtained. In terms of α-diversity, both fecal and rumen fluid microbiota were not affected significantly by FPPR (*p* > 0.05). Separation among the CON, B25, and B50 fecal samples was not noticeable (Figure 4Ab,d). However, the result of the analysis of similarities (ANOSIM) (Table 4) revealed that the *R*-values of CON/B25, CON/B50, and B25/B50 were all greater than 0, indicating that the differences between the groups were significantly greater than those within the groups. Furthermore, in this study, the *p*-values for CON/B50 and B25/B50 were less than 0.05, demonstrating that there were significant differences between the two treatments. The results of principal co-ordinates analysis (PCoA) and nonmetric multidimensional scaling (NMDS) for the β-diversity of rumen fluid (Figure 4Bb,d) revealed a noticeable separation between CON and B50. The ANOSIM results (Table 4) unanimously showed that the *R*-value of CON/B50 was greater than 0 and the *p*-value of CON/B50 was less than 0.05. These results indicated that FPPR significantly affected the β-diversity of rumen fluid microbiota.

**Table 4:** Analysis of similarities (ANOSIM) of the composition and predicted function in fecal and rumen fluid microflora.

**Item**		**Microflora**		**Predicted function**	
		***R*-value**	***P*-value**	***R*-value**	***P*-value**
Feces	CON-B25	0.0993	0.055	0.0614	0.136
	CON-B50	0.1588	0.047	0.0915	0.106
	B25-B50	0.1828	0.012	−0.0530	0.739
Rumen Fluid	CON-B50	0.4446	0.001	0.0357	0.265

### The composition of fecal and rumen fluid microbiota

**Figure 5:**
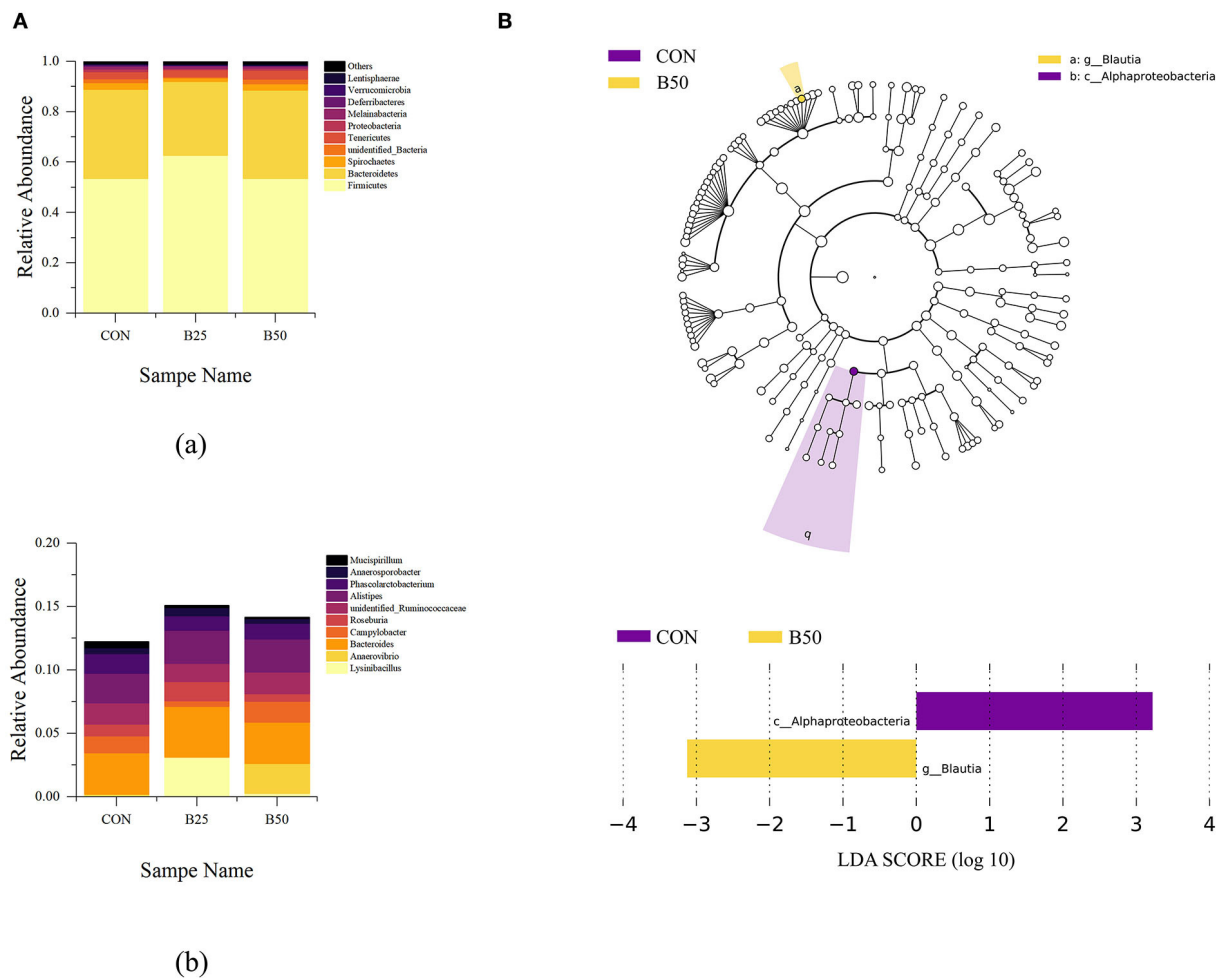
Composition and linear discriminant analysis effect size (LEfSe) analyses of fecal microflora. **(A)** Component of fecal microbiota: (a) phylum level; (b) Genus level. **(B)** LEfSe analysis plot of fecal microflora.

The effects of FPPR on fecal microflora are shown in Figure 5A. *Firmicutes, Bacteroidetes, Spirochaetes, unidentified bacteria, Tenericutes, Proteobacteria, Melainabacteria, Deferribacteres, Verrucomicrobia, and Lentisphaerae* were the most abundant bacteria at the phylum level. *Lysinibacillus, Anaerovibrio, Bacteroides, Campylobacter, Roseburia, unidentified Ruminococcaceae, Alistipes, Phascolarctobacterium, Anaerosporobacter*, and *Mucispirillum* were the dominant bacteria at the genus level. Among the dominant phyla, significant differences were found in Firmicutes and Bacteroidetes. In addition, among the dominant genera, *Lysinibacillus* had significantly greater abundance in B25 than in CON and B50. LEfSe analysis is an analytical tool for discovering and interpreting high-dimensional biomarkers in microbial research. It emphasizes statistical significance and biological correlation and can be used to find biomarkers with statistical differences among groups. Two differential OTUs were identified in fecal samples under the three treatments (Figure 5B), including one genus and one class that were each distributed in CON and B50. B25 had no biomarker. The LDA score plot showed that Alphaproteobacteria was upregulated and obviously enriched in CON, whereas *Blautia* was downregulated and obviously enriched in B50.

**Figure 6:**
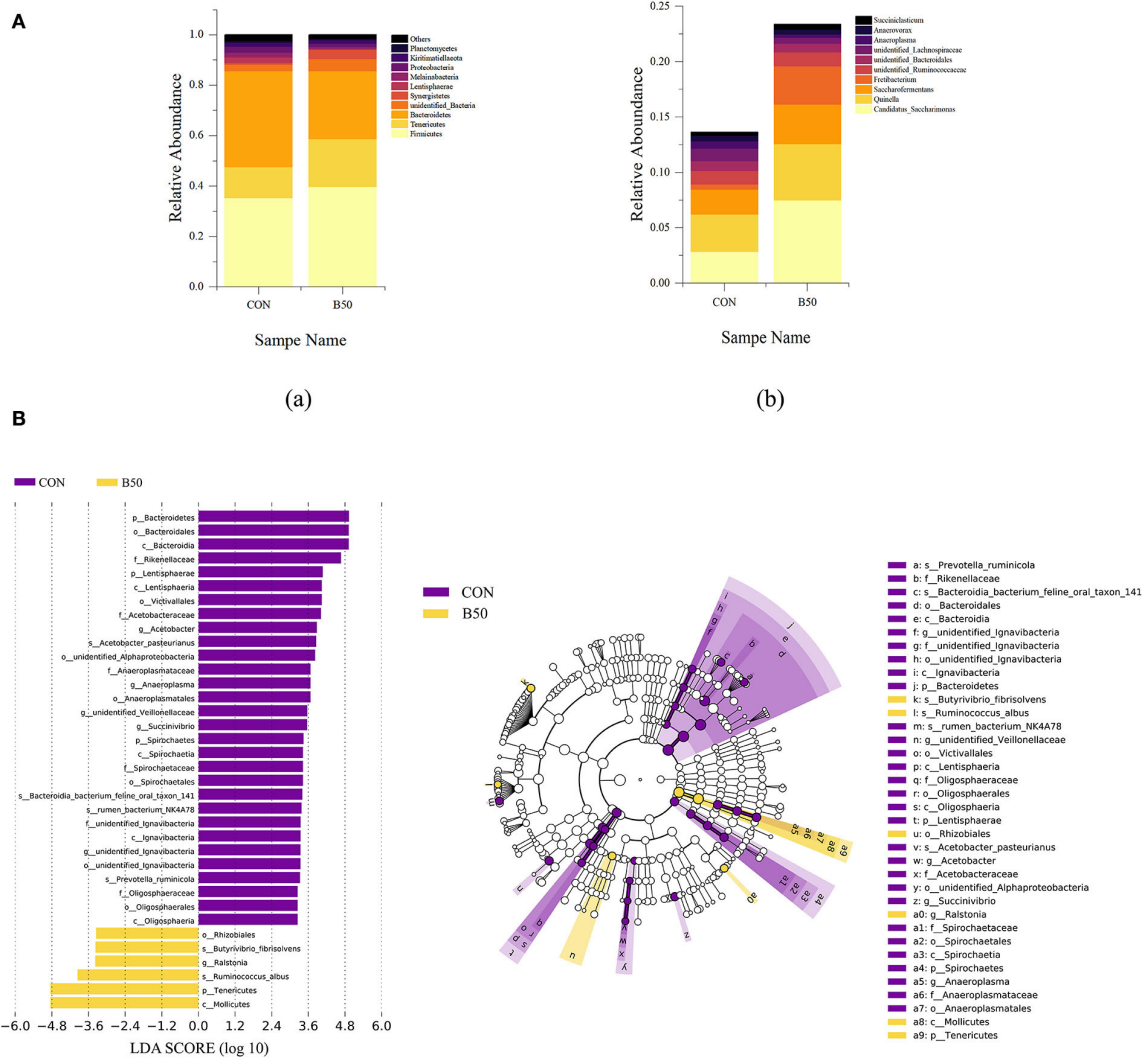
Composition and LEfSe analyses of rumen fluid microflora. **(A)** Component of rumen fluid microbiota: (a) phylum level; (b) genus level. **(B)** LEfSe analysis plot of rumen fluid microflora.

The composition of rumen fluid in CON and B50 are shown in Figure 6A. At the phylum level, the dominant bacteria were *Firmicutes, Tenericutes, Bacteroidetes, unidentified_bacteria, Synergistetes, Lentisphaerae, Melainabacteria, Proteobacteria, Kiritimatiellaeota, and Planctomycetes*. Compared to CON, the relative abundance of Bacteroidetes had decreased in B50 (*p* < 0.05). At the genus level, the dominant bacteria were *Candidatus_Saccharimonas, Quinella, Saccharofermentans, Fretibacterium, unidentified_Ruminococcaceae, unidentified_Bacteroidales, unidentified_Lachnospiraceae, Anaeroplasma, Anaerovorax*, and *Succiniclasticum*. The microflora compositions of CON and B50 did not significantly differ. In addition, LEfSe analyses found 26 differential OTUs between CON and B50 (Figure 6B). Among these OTUs, six were upregulated and 30 were downregulated in B50.

### Predicted function of fecal and rumen fluid microflora

**Figure 7:**
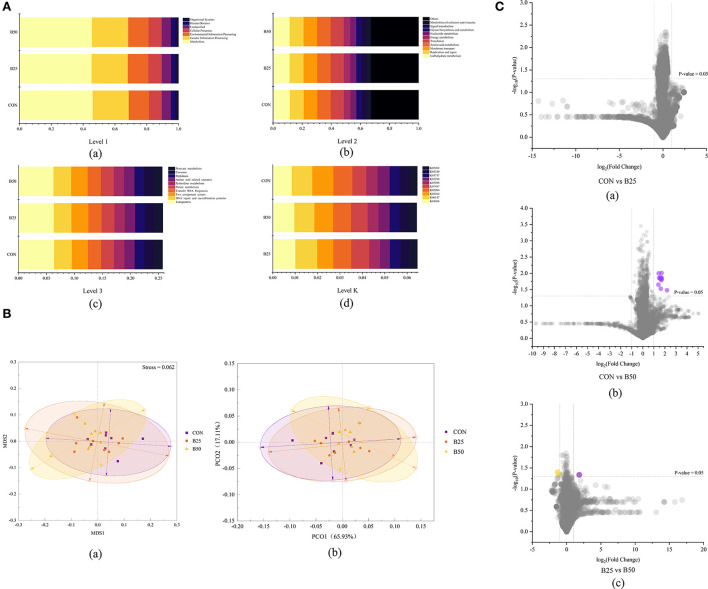
Functional prediction on the composition and difference for fecal microflora in the three treatments. **(A)** Functional prediction on the fecal microflora: (a–d) bar plot of the relative abundance of the predicted function on levels 1, 2, 3, and K; **(B)** (a) NMDS plot; (b) PCoA plot; and **(C)** pairwise comparison of different functions between the groups based on the *t*-test.

In this study, 6,043 Kyoto Encyclopedia of Genes and Genomes (KEGG) orthologs in fecal microflora were predicted by Tax4Fun. In the first level, these KEGG orthologs were related to organismal systems, human diseases, cellular process, environmental information processing, genetic information processing, and metabolism (Figure 7Aa). In the second level, fecal microflora were connected with the metabolism of cofactors and vitamins, signal transduction, glycan biosynthesis and metabolism, carbohydrate metabolism, and replication and repair (Figure 7Ab). Figure 7Ac shows that at the third level, the dominant pathways were transporters, DNA repair and recombination proteins, a two-component system, transfer ribonucleic acid (RNA) biogenesis, and purine metabolism. Figure 7Ad illustrates that the top 5 KEGG orthologs were K03406, K06147, K02014, K02004, and K07497. The β-diversity of the predicted functions in feces was determined after pathway annotation (Figure 7B). The PCoA and NMDS score plots revealed no obvious separations among the three treatments. ANOSIM analyses were also performed and, as shown in Table 4, no significant difference was found among the three treatments (*p* > 0.05). The results indicated that FPPR had no effect on the function of fecal microflora. The difference analysis results of functional prediction are provided in Figure 7C. Volcano plots (*p* < 0.05, fold-change = 2) showed 12 upregulated points in CON–B25 and one upregulated and two downregulated points in B25–B50. Different KEGG orthologs are given in Table 5.

**Table 5:** Significant differential KEGG orthologs of functional prediction in fecal microorganisms.

	**KEGG orthologs**	**Fold change**	***p*-value**
CON vs. B50	K00204	3.11	0.01
	K03231	3.24	0.01
	K03573	3.09	0.03
	K03626	3.28	0.01
	K05576	3.26	0.02
	K06011	2.9	0.01
	K07730	3.06	0.02
	K09835	2.67	0.02
	K11005	2.81	0.01
	K11058	2.72	0.01
	K14121	4.55	0.03
	K15896	3.19	0.02
B25 vs. B50	K07445	0.45	0.05
	K08097	0.4	0.04
	K02077	3.6	0.05

**Figure 8:**
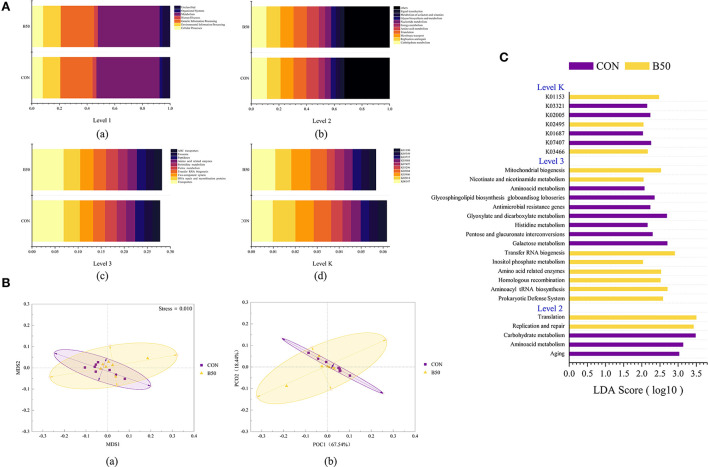
Functional prediction on the composition and LEfSe analyses for rumen fluid microflora affected by FPPR: **(A)** Functional prediction on fecal microflora: (a–d) Bar plot of the relative abundance of the predicted function on Levels 1, 2, 3, and K; **(B)** (a) NMDS plot; (b) PCoA plot; and **(C)** LAD bar plot for the predicted function of rumen microflora.

Tax4Fun detected 6,278 KEGG orthologs in rumen fluid. The first level (Figure 8Aa) that was related to rumen fluid microflora included cellular processes, environmental information processing, genetic information processing, human diseases, metabolism, organismal systems, and unclassified. In the second level (Figure 8Ab), the primary orthologs in rumen fluid were related to carbohydrate metabolism, replication and repair, membrane transport, translation, and amino acid metabolism. Figure 8Ac shows that at the third level, the dominant orthologs were transporters, DNA repair and recombination proteins, a two-component system, transfer RNA biogenesis, and purine metabolism. The main orthologs at the K level (Figure 8Ad) were K06147, K02014, K03406, K02004, and K03296. The β-diversity results (Figure 8B) from PCoA and NMDS analyses revealed no noticeable separation between CON and B50. The ANOSIM results given in Table 4 further illustrated the lack of differentiation (*p* > 0.05). In addition, LEfse analysis showed that 27 differential pathways were found in rumen fluid microflora (Figure 8C). Of these pathways, 14 and 13 were significantly enriched in CON and B50, respectively.

## Discussion

Fermented pineapple peel residue is an unconventional feedstuff (UCF) with the characteristics of unique fragrance, sufficient water content, soft texture, and good palatability. It can considerably stimulate the appetite of black goats. This study showed that FPPR had an obvious effect on the weight gain of black goats. In addition, as the experimental period proceeded, the regularity of the feed intake of the goats in the FPPR group increased, indicating that FPPR might lessen the susceptibility of goats to the adverse influence of external factors.

Various biochemical components in the blood are the material basis of life activities, and their contents and changes reflect the basic situation of nutrition and metabolism in the body. UREA is the decomposition product of protein and fat and plays an important role in renal function (Liu et al., 2020; Lu et al., 2020). A previous research showed that high UREA reflects the high decomposition rates of proteins and amino acids and indicates the levels of fatigue in the body (Wang et al., 2020). High levels of NEFA in the blood are often used as a sign of negative energy balance or fat mobilization (Hendriks et al., 2022). Compared with WCS without FPPR, the partially replaced feeds contained more energy that can be provided to goats and were more beneficial to goat fat deposition during production. In this study, other biochemical indicators did not show no significant differences between the two treatments, indicating that the replacement of WCS with FPPR had no adverse effect on the physical status of black goats.

The α-diversity analysis is a single-sample microflora diversity test based on OTU clustering results. Biodiversity can be estimated by calculating species richness indexes, such as the Chao1 index, and diversity indexes, such as Shannon index. In this study, FPPR did not appear to cause significant changes in the abundance or diversity of microorganisms in rumen fluid or feces (*p* > 0.05). PCoA and NMDS can reflect the variance within and between the groups based on the distance between dots. Furthermore, ANOSIM is a nonparametric test that is performed to determine the presence of significant differences in the community structure between the groups and to compare between- and within-group differences. Using a combination of the three analysis methods mentioned earlier can provide a great indication of the β-diversity of the microbiota. In this study, the different levels of FPPR addition did not cause significant differences in fecal microflora (*p* > 0.05). However, LEfse analysis showed that *g_Blautia* and c_Alphaproteobacteria that differed significantly in each treatment were enriched in B50 and CON, respectively. Previous studies have demonstrated that *Blautia* is a group of beneficial microorganisms that can be beneficial to glucose metabolism and obesity-associated inflammation (Ding et al., 2022). Functional prediction of the microflora did not significantly differ between the treatments with or without FPPR addition, indicating that FPPR presumably did not cause changes in microbial functions.

Furthermore, goats that were highly affected by FPPR in terms of ADG and feed intake were selected to further explore the effects of partially replacing WCS with FPPR on Chuanzhong black goats. Then, the effects of FPPR on rumen microflora and meat quality were studied after the goats were slaughtered.

Muscle pH is related to the glycolysis of glycogen and plays an important role in meat quality (Beauclercq et al., 2016). Studies have reported that when animals are in a highly stressed state before slaughter, the pH of the muscles increases significantly (El Otmani et al., 2021; Zhang J. Y. et al., 2021). The results indicated that in black goats, FPPR might reduce preslaughter stress, but had no effect on the flesh color, tenderness, and WHC of longissimus dorsi. For amino acid composition, the content of phenylalanine under the FPPR treatment was lower than that under CON. However, TAAs, EAAs, and DAA did not significantly differ between CON and B50. Thus, FPPR had little effect on the amino acid composition.

The rumen is vital for ruminant digestion and health. It contains microbes that enable ruminants to digest plant fiber for energy, and this environment is a natural home for many anaerobic bacteria. Moreover, rumen fermentation provides 70% of a ruminant's energy (Fregulia et al., 2021). *s_Butyrivibrio fibrisolvens, s_Ruminococcus albus, g_Ralstonia, o_Rhizobiales, c_Mollicutes*, and *p_Tenericutes* were affected by FPPR and became biomarkers and enriched in B50. *s_Butyrivibrio fibrisolvens* and *s_Ruminococcus albus* are bacteria that are good for ruminants. *s_Butyrivibrio fibrisolvens* can increase the content of conjugated linoleic acid in the intestine and adipose tissue (Srivastava et al., 2021) and produce diverse carbohydrate-active enzymes that hydrolyze cellulose and other plant-derived macromolecular polymers (Sengupta et al., 2022). This bacterium is similar to *s_Ruminococcus albus*, which can also synthesize some types of hydrolytic enzymes that help ruminants digest plant-based feed (Storani et al., 2020; Ortiz-Chura et al., 2021). Firmicutes and Bacteroidetes are essential for ruminant digestion. Bacteroidetes plays an important role in the degradation of nonfibrous and protein materials, whereas Firmicutes is mainly involved in the degradation of fibrous materials (Zhang X. et al., 2021). These two phyla were the most abundant phyla in the rumen of goats in this experiment, but were not significantly enriched in either group, likely because the partial substitution of Water Soluble Carbohydrates (WSC) by FPPR did not result in an obvious difference in fiber and protein content and, therefore, did not cause any significant differences between the two bacterial phyla. Similar to this experiment, a previous work found that Tenericutes, the third most abundant phylum in the rumen and the phylum that was enriched in the group under FPPR treatment, has a significant association with feed intake (Zhang Y. K. et al., 2021). Nevertheless, other studies have shown that Tenericutes is abundant in animals at high risk of subacute ruminal acidosis (Li et al., 2017). However, no symptomatic goats were found in this study. This finding will be the focus of further research on the use of FPPR to replace WCS. The results shown above indicated that although replacing WCS with FPPR can increase the number of bacteria that are capable of degrading and increasing the ADG of goats, it did not cause differences between the two main phyla in the rumen and can likely improve the digestion ability of the rumen to some extent in Chuanzhong black goats. Additionally, *g_Ralstonia* can be used to degrade monofluoroactetate (MFA) contained in plants, and in goats, the inoculation of *g_Ralstonia* into the rumen under artificial conditions can effectively relieve the symptoms of MFA poisoning (da Silva et al., 2016). Among the predicted functions affected by FPPR, pathways related to replication and repair, translation, and K03466 (ftsK, DNA segregation ATPase FtsK/SpoIIIE) were enriched. Metabolism-related functions, such as inositol phosphate metabolism, amino-acid-related enzymes, nicotinate, and nicotinamide metabolism, were also enriched by FPPR replacement. The abovementioned phenomena indicated that the mixed feed might have promoted the proliferation and metabolic activity of bacteria.

## Conclusion

This study revealed that FPPR has potential as a substitute for WCS. Like UCF, FPPR had no harmful effects on the physical status of Chuanzhong black goats. In contrast, feed intake and ADG improved. Replacing WCS with a certain ratio of FPPR might play a positive role in the hindgut and rumen health manifested as the upregulation of probiotics, such as *Blautia, B. fibrisolvens*, and *R. albus*. FPPR could exert significant effects on the composition of fecal and rumen microflora. However, the mechanism underlying these effects is unclear and requires deep research.

## Data availability statement

The raw sequence data reported in this paper have been deposited in the Genome Sequence Archive (Genomics, Proteomics & Bioinformatics 2021) in National Genomics Data Center (Nucleic Acids Res 2022), China National Center for Bioinformation / Beijing Institute of Genomics, Chinese Academy of Sciences (GSA: CRA007078 and CRA007079) that are publicly accessible at https://ngdc.cncb.ac.cn/gsa.

## Ethics statement

The animal study was reviewed and approved by the Committee of Animal Experiments of South China Agricultural University.

## Author contributions

CY, WZ, BS, CG, and MW conceived and designed the study. CY, HT, WZ, YG, MW, and CG performed the experiments. MW and YG organized the database and performed the statistical analysis. CY and HT wrote the manuscript. HT and WZ visualized the results. YG and BS revised this manuscript. All authors contributed to the article and approved the submitted version.

## Funding

This research has received funding from the Modern Agricultural Industrial Technology System of Guangdong province (2022KJ127), Guangdong Basic and Applied Basic Research Foundation of China (2019B1515210020), and Key-Area Research and Development Program of Guangdong province (2019B110209005).

## Conflict of interest

The authors declare that the research was conducted in the absence of any commercial or financial relationships that could be construed as a potential conflict of interest.

## Publisher's note

All claims expressed in this article are solely those of the authors and do not necessarily represent those of their affiliated organizations, or those of the publisher, the editors and the reviewers. Any product that may be evaluated in this article, or claim that may be made by its manufacturer, is not guaranteed or endorsed by the publisher.
